# Supplementary material for: Characterizing the role of early life factors in machine learning-based multimorbidity risk prediction
Source: PLOS Digit Health. 2025 Aug 18;4(8):e0000982. doi: 10.1371/journal.pdig.0000982 (PMC12360575; doi:10.1371/journal.pdig.0000982)
Supplement: S1 Table — (PDF) [file pdig.0000982.s001.pdf]

S1 Table: Summary of all UKB input variables used in the study.

**Category**	**Variables**
Early-life factors	BreastfedAsABaby, AdoptedAsAChild, MaternalSmokingAroundBirth, FeltHatedByFamilyMemberAsAChild, PhysicallyAbusedByFamilyAsAChild, FeltLovedAsAChild, SexuallyMolestedAsAChild, SomeoneToTakeToDoctorWhenNeededAsAChild<br>*Number of Variables: 8*
Sociodemographics	Sex, EthnicBackground, Age, Qualifications, CurrentEmploymentStatus, AvgHouseholdIncome<br>*Number of Variables: 6*
Physical Measures & Activity	WaistCircumference, BMI, BodyFatPercentage, DiastolicBloodPressure, SystolicBloodPressure, SleepDuration, IPAQActivityGroup<br>*Number of Variables: 7*
Diet and Nutrition	CookedVegetableIntake, FreshFruitIntake, OilyFishIntake, ProcessedMeatIntake, BeefIntake, PorkIntake, SaltAddedToFood, WaterIntake, RawVegetableIntake, CerealIntake, PoultryIntake<br>*Number of Variables: 11*
Substance Use	CurrentTobaccoSmoking, SmokingStatus, AlcoholDrinkerStatus, AlcoholIntakeFrequency<br>*Number of Variables: 4*
Health and Medical History	OverallHealthRating, CancerDiagnosedByDoctor, Diabetes (CVD), Depression, Anxiety, Hypertension<br>*Number of Variables: 6*
Psychosocial Factors	MoodSwings, Irritability, LonelinessIsolation, LeisureSocialActivities<br>*Number of Variables: 4*
Family History	IllnessesOfFather (CVD, Depression, Diabetes), IllnessesOfMother (CVD, Depression, Diabetes), IllnessesOfSiblings (CVD, Depression, Diabetes)<br>*Number of Variables: 9*
Blood Assays	ApolipoproteinA, ApolipoproteinB, Cholesterol, CReactiveProtein, Glucose, HbA1c, HDLCholesterol, LDLDirect, Triglycerides<br>*Number of Variables: 9*
	**Total Number of Variables: 64**
