# Supplementary material for: Characterizing the role of early life factors in machine learning-based multimorbidity risk prediction
Source: PLOS Digit Health. 2025 Aug 18;4(8):e0000982. doi: 10.1371/journal.pdig.0000982 (PMC12360575; doi:10.1371/journal.pdig.0000982)
Supplement: S1 Fig — (PDF) [file pdig.0000982.s002.pdf]

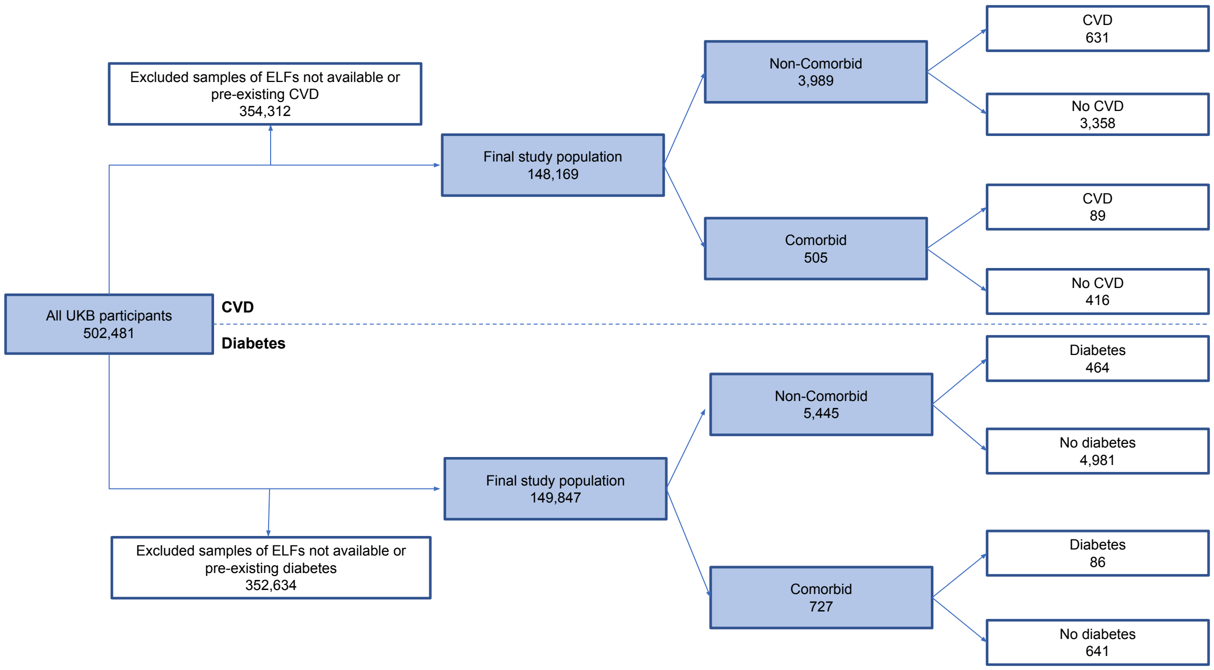

S1 Figure: Flow diagram of participant stratification based on early-life factors (ELFs), detailing CVD and diabetes outcomes in non-comorbid and comorbid subgroups.
