# Supplementary material for: Characterizing the role of early life factors in machine learning-based multimorbidity risk prediction
Source: PLOS Digit Health. 2025 Aug 18;4(8):e0000982. doi: 10.1371/journal.pdig.0000982 (PMC12360575; doi:10.1371/journal.pdig.0000982)
Supplement: S2 Table — (PDF) [file pdig.0000982.s003.pdf]

S2 Table: Permutation-based variable ranking for predicting CVD in non-comorbid and comorbid groups.

**Variable (Non-Comorbid)**	**Score**	**Variable (Comorbid)**	**Score**
Age*	0.081893	Age*	0.056253
Sex*	0.022991	OverallHealthRating	0.016420
Hypertension*	0.007651	Glucose	0.007265
OverallHealthRating	0.007617	CurrentEmploymentStatus_Retired	0.007114
WaistCircumference	0.003830	WaistCircumference	0.006805
SystolicBloodPressure*	0.003604	HbA1c	0.006483
ApolipoproteinB	0.001830	CurrentEmploymentStatus_Paid/SelfEmployed	0.003173
HDLCholesterol	0.001107	LDLDirect	0.003124
CurrentEmploymentStatus_Paid/SelfEmployed	0.000702	IllnessesOfMother_CVD	0.002986
CReactiveProtein	0.000623	SmokingStatus*	0.002780
SmokingStatus*	0.000586	DiastolicBloodPressure	0.002697
ApolipoproteinA	0.000540	Depression	0.002218
IllnessesOfSiblings_CVD	0.000495	CurrentEmploymentStatus_Sick/Disabled	0.002155
HbA1c	0.000491	PorkIntake	0.001957
BodyFatPercentage	0.000444	CReactiveProtein	0.002057
CancerDiagnosedByDoctor	0.000362	PhysicallyAbusedByFamilyAsAChild	0.002031
Triglycerides	0.000347	Cholesterol	0.001815
DiastolicBloodPressure	0.000294	ApolipoproteinA	0.001792
LDLDirect	0.000277	BodyFatPercentage	0.001593
CurrentTobaccoSmoking	0.000270	MaternalSmokingAroundBirth	0.001591
FeltHatedByFamilyMemberAsAChild	0.000255	SaltAddedToFood	0.001431
Cholesterol	0.000171	SleepDuration	0.001137
AvgHouseholdIncome	0.000166	AlcoholIntakeFrequency	0.000868
IllnessesOfFather_CVD	0.000155	WaterIntake	0.000839
SomeoneToTakeToDoctorWhenNeededAsAChild	0.000148	FeltHatedByFamilyMemberAsAChild	0.000759
Glucose	0.000123	BreastfedAsABaby	0.000708
Qualifications_NVQ/HND/HNC	0.000118	Qualifications_UnivDegree	0.000623
HDLCholesterol	0.001107	HDLCholesterol	0.000580
IllnessesOfMother_CVD	0.000096	MoodSwings	0.000530
CurrentEmploymentStatus_Sick/Disabled	0.000089	Qualifications_ProfQual(Nurse/Teach)	0.000517

*Variables employed in current risk assessment models.
