# Supplementary material for: Characterizing the role of early life factors in machine learning-based multimorbidity risk prediction
Source: PLOS Digit Health. 2025 Aug 18;4(8):e0000982. doi: 10.1371/journal.pdig.0000982 (PMC12360575; doi:10.1371/journal.pdig.0000982)
Supplement: S3 Table — (PDF) [file pdig.0000982.s005.pdf]

S3 Table: Permutation-based variable ranking for predicting diabetes in non-comorbid and comorbid groups.

**Variable (Non-Comorbid)**	**Score**	**Variable (Comorbid)**	**Score**
HbA1c	0.093880	HbA1c	0.119222
WaistCircumference*	0.019956	WaistCircumference*	0.023598
Glucose	0.009616	OverallHealthRating	0.010247
BMI*	0.004360	Triglycerides	0.006574
Hypertension*	0.002537	HDLCholesterol	0.005897
Triglycerides	0.002440	Glucose	0.003622
HDLCholesterol	0.002029	Hypertension*	0.003426
OverallHealthRating	0.001623	Sex	0.003144
AlcoholIntakeFrequency	0.001076	BodyFatPercentage	0.002629
IllnessesOfMother_Diabetes*	0.000981	Age*	0.001551
Cholesterol	0.000946	CurrentEmploymentStatus_Sick/Disabled	0.001472
Age*	0.000916	Qualifications_UnivDegree	0.001422
CReactiveProtein	0.000545	BMI*	0.001389
AvgHouseholdIncome	0.000536	CerealIntake	0.000909
BodyFatPercentage	0.000404	SomeoneToTakeToDoctorWhenNeededAsAChild	0.000909
ApolipoproteinA	0.000390	FeltHatedByFamilyMemberAsAChild	0.000865
IllnessesOfSiblings_Diabetes*	0.000381	SleepDuration	0.000858
LDLDirect	0.000346	AvgHouseholdIncome	0.000729
SystolicBloodPressure	0.000340	RawVegetableIntake	0.000705
Sex	0.000321	ProcessedMeatIntake	0.000691
ApolipoproteinB	0.000318	Depression	0.000648
EthnicBackground_White*	0.000280	Qualifications_NoneAbove	0.000625
IllnessesOfFather_Diabetes*	0.000252	IllnessesOfMother_Diabetes*	0.000591
WaterIntake	0.000166	IllnessesOfSiblings_Diabetes*	0.000532
LonelinessIsolation	0.000130	CReactiveProtein	0.000442
Qualifications_UnivDegree	0.000117	IllnessesOfFather_CVD	0.000434
CurrentEmploymentStatus_Student	0.000106	FeltLovedAsAChild	0.000416
BreastfedAsABaby	0.000104	AlcoholIntakeFrequency	0.000394
ProcessedMeatIntake	0.000103	PoultryIntake	0.000387
DiastolicBloodPressure	0.000101	IllnessesOfMother_CVD	0.000347

*Variables employed in current risk assessment models.
