# Supplementary material for: Characterizing the role of early life factors in machine learning-based multimorbidity risk prediction
Source: PLOS Digit Health. 2025 Aug 18;4(8):e0000982. doi: 10.1371/journal.pdig.0000982 (PMC12360575; doi:10.1371/journal.pdig.0000982)
Supplement: S3 Fig — (b) Proportion of early-life factors, underlining their frequency and potential impact on later diabetes health outcomes. (PDF) [file pdig.0000982.s006.pdf]

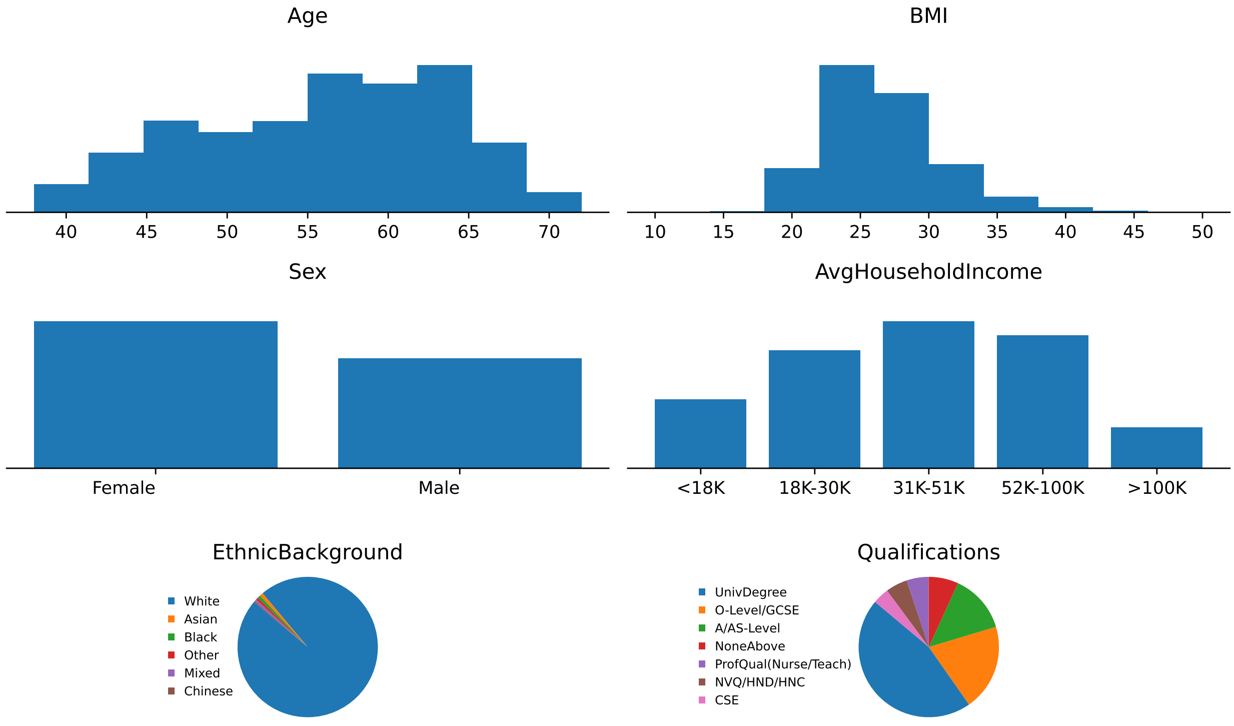

(a) Participant demographic and socioeconomic metrics.

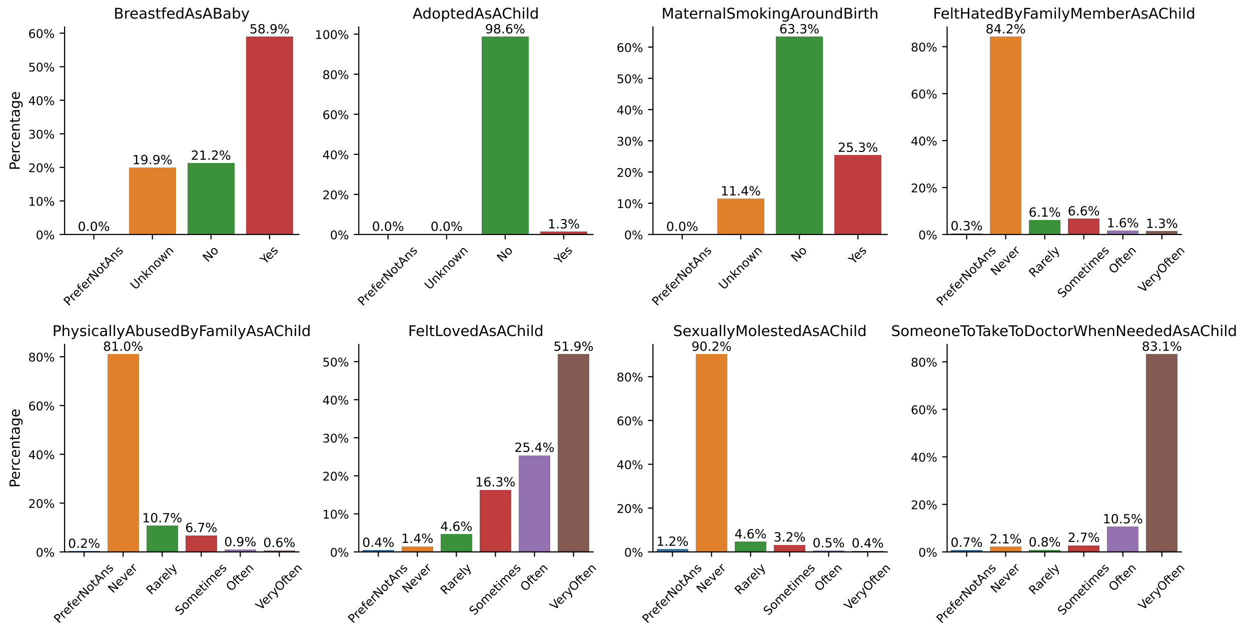

(b) Early-life factor prevalence.

S3 Figure: Comprehensive overview of participant profiles for the final study cohort with diabetes outcomes: (a) Demographic and socioeconomic distribution, illustrating the population sample's diversity. (b) Proportion of early-life factors, underlining their frequency and potential impact on later diabetes health outcomes.
