# Supplementary material for: Characterizing the role of early life factors in machine learning-based multimorbidity risk prediction
Source: PLOS Digit Health. 2025 Aug 18;4(8):e0000982. doi: 10.1371/journal.pdig.0000982 (PMC12360575; doi:10.1371/journal.pdig.0000982)
Supplement: S4 Table — The top 30 features are ranked by their effect size for CVD. (PDF) [file pdig.0000982.s007.pdf]

S4 Table: Differential top features by effect size: comorbid vs. non-comorbid. The top 30 features are ranked by their effect size for CVD.

**Variable (Non-Comorbid)**	**AvgRank**	**Variable (Comorbid)**	**AvgRank**
Age*	2.2	Age*	2
WaistCircumference	3	CurrentEmploymentStatus_Retired	4.2
Hypertension*	3.8	CurrentEmploymentStatus_Paid/SelfEmployed	4.4
CurrentEmploymentStatus_Retired	3	OverallHealthRating	7.2
CurrentEmploymentStatus_Paid/SelfEmployed	6	Hypertension*	7.4
Sex*	6.4	Sex*	8.9
SystolicBloodPressure*	6.8	WaistCircumference	14.2
OverallHealthRating	7.4	Cholesterol	15.8
HDLCholesterol	9.8	LDLDirect	19
BMI*	10	AvgHouseholdIncome	21.2
ApolipoproteinA	11.8	HDLCholesterol	24.6
AvgHouseholdIncome	12	Glucose	25.2
Triglycerides	13	BMI*	25.8
HbA1c	13.4	MoodSwings	26
DiastolicBloodPressure	15	FeltLovedAsAChild	26.8
Qualifications_NoneAbove	17.6	HbA1c	26.8
SmokingStatus*	18.8	AlcoholIntakeFrequency	29.2
CReactiveProtein	20.8	CancerDiagnosedByDoctor	29.6
ProcessedMeatIntake	21.6	ApolipoproteinB	31
WaterIntake	25.2	ApolipoproteinA	32.2
Glucose	26	IllnessesOfSiblings_CVD	32.3
Qualifications_UnivDegree	26.8	BreastfedAsABaby	34.6
LeisureSocialActivities_SportsClubOrGym	27.2	CurrentEmploymentStatus_Unemployed	35.4
LeisureSocialActivities_PubOrSocialClub	28.2	SystolicBloodPressure*	35.8
LDLDirect	28.4	DiastolicBloodPressure	36.2
Cholesterol	29	Qualifications_NVQ/HND/HNC	36.2
PorkIntake	29.2	CookedVegetableIntake	37.2
BreastfedAsABaby	30.6	FeltHatedByFamilyMemberAsAChild	37.2
IllnessesOfSiblings_CVD	31	Qualifications_ProfQual(Nurse/Teach)	38.1
BeefIntake	31.2	SexuallyMolestedAsAChild	38.4

*Variables employed in current risk assessment models.
