# Supplementary material for: Characterizing the role of early life factors in machine learning-based multimorbidity risk prediction
Source: PLOS Digit Health. 2025 Aug 18;4(8):e0000982. doi: 10.1371/journal.pdig.0000982 (PMC12360575; doi:10.1371/journal.pdig.0000982)
Supplement: S4 Fig — Complements Fig 3 by showing the full distribution of AUC-ROC values for all features. (PDF) [file pdig.0000982.s008.pdf]

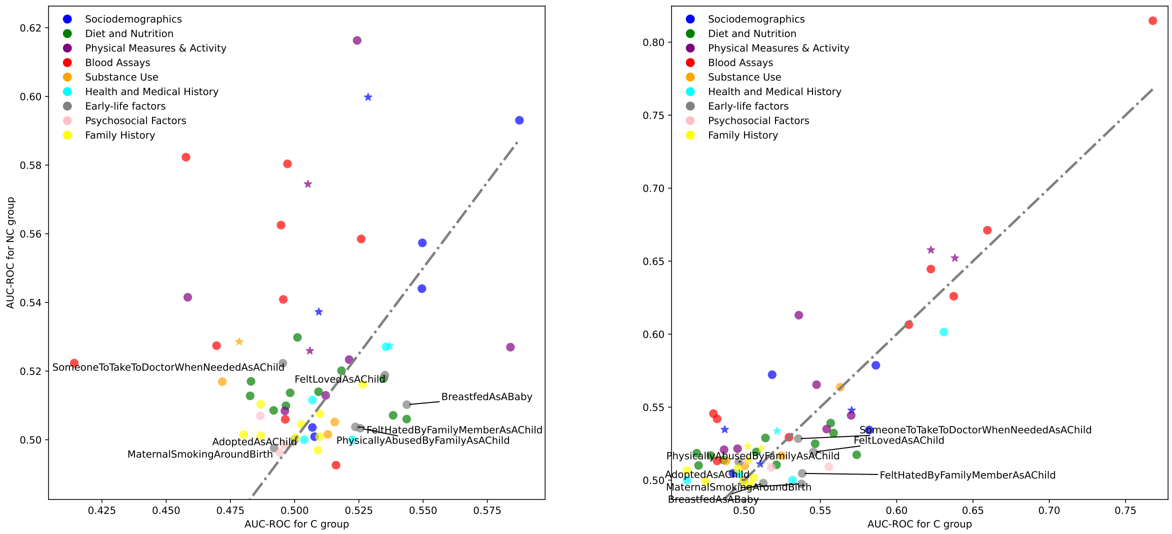

(a) XGB CVD

(b) XGB Diabetes

S4 Figure: Predictive ability of individual variables for (a) CVD and (b) diabetes prediction. Each plot compares AUC-ROC performance in the comorbid group (x-axis) vs performance in the non-comorbid (y-axis) group. Each point represents an XGB model learned with a single feature. Colors differentiate models learned with a variable of different type. The star marker indicates models learned with a variable used by the benchmarks. The dotted grey line indicates equal performance in both groups. Deviation from this line allows for identifying variables that have a stronger predictive impact on one group over the other.
