# Supplementary material for: Characterizing the role of early life factors in machine learning-based multimorbidity risk prediction
Source: PLOS Digit Health. 2025 Aug 18;4(8):e0000982. doi: 10.1371/journal.pdig.0000982 (PMC12360575; doi:10.1371/journal.pdig.0000982)
Supplement: S5 Table — The top 30 features are ranked by their effect size for diabetes. (PDF) [file pdig.0000982.s009.pdf]

S5 Table: Differential top features by effect size: comorbid vs. non-comorbid. The top 30 features are ranked by their effect size for diabetes.

**Variable (Non-Comorbid)**	**AvgRank**	**Variable (Comorbid)**	**AvgRank**
HbA1c	1.4	HbA1c	1.2
Glucose	1.6	WaistCircumference*	4.8
WaistCircumference*	3.2	BMI*	4.8
BMI*	3.8	Triglycerides	13.4
Triglycerides	5	Glucose	13.8
HDLCholesterol	6.2	CReactiveProtein	14
CReactiveProtein	7.4	HDLCholesterol	18
ApolipoproteinA	8.4	AlcoholDrinkerStatus	20.8
OverallHealthRating	10.6	Depression	21.2
DiastolicBloodPressure	11	Anxiety	21.4
SystolicBloodPressure	11	ApolipoproteinA	23.8
Hypertension*	11.2	OverallHealthRating	24.8
BodyFatPercentage	12	IllnessesOfSiblings_CVD	26.1
Sex*	14.8	Irritability	26.6
AlcoholIntakeFrequency	18.6	AlcoholIntakeFrequency	27.2
IllnessesOfSiblings_Diabetes*	19	SmokingStatus	27.2
ApolipoproteinB	20.2	SexuallyMolestedAsAChild	28.2
ProcessedMeatIntake	20.8	Qualifications_O-Level/GCSE	28.2
IPAQActivityGroup	21.2	EthnicBackground_White*	28.4
AvgHouseholdIncome	21.6	SleepDuration	28.4
EthnicBackground_White*	24	BodyFatPercentage	29.2
LeisureSocialActivities_SportsClubOrGym	24.4	IllnessesOfFather_Diabetes*	29.4
IllnessesOfMother_Diabetes*	25.4	IllnessesOfSiblings_Diabetes*	29.8
Age*	27.4	Hypertension*	33.8
SmokingStatus	27.6	IllnessesOfMother_Diabetes*	34
IllnessesOfFather_Diabetes*	28.4	Qualifications_ProfQual(Nurse/Teach)	34.1
CurrentEmploymentStatus_Sick/Disabled	31.6	FreshFruitIntake	34.2
CerealIntake	31.8	MaternalSmokingAroundBirth	34.2
PorkIntake	35.2	LeisureSocialActivities_NoneAbove	34.4
Qualifications_NoneAbove	35.4	CurrentEmploymentStatus_Paid/SelfEmployed	36

*Variables employed in current risk assessment models.
