# Supplementary material for: Characterizing the role of early life factors in machine learning-based multimorbidity risk prediction
Source: PLOS Digit Health. 2025 Aug 18;4(8):e0000982. doi: 10.1371/journal.pdig.0000982 (PMC12360575; doi:10.1371/journal.pdig.0000982)
Supplement: S6 Fig — Variables marked with an asterisk ( ) are those currently employed in current risk assessment models. (PDF) [file pdig.0000982.s011.pdf]

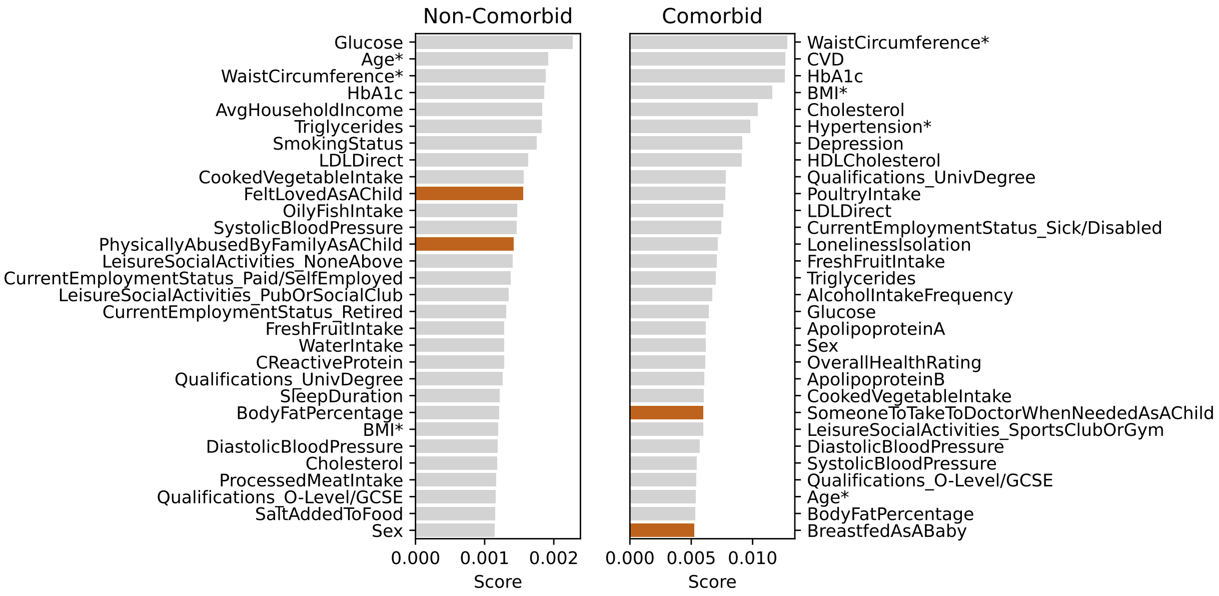

S6 Figure: SHAP scores of the top 30 influential variables in the diabetes prediction model for non-comorbid and comorbid groups. Variables marked with an asterisk (*) are those currently employed in existing risk assessment models.
