# Supplementary material for: Characterizing the role of early life factors in machine learning-based multimorbidity risk prediction
Source: PLOS Digit Health. 2025 Aug 18;4(8):e0000982. doi: 10.1371/journal.pdig.0000982 (PMC12360575; doi:10.1371/journal.pdig.0000982)
Supplement: Attachment — Submitted filename: Response to Reviewers.pdf [file pdig.0000982.s012.pdf]

RESPONSE LETTER TO THE REVIEWERS' COMMENTS

Ms. Ref. No.: PDIG-D-25-00125

Characterizing the role of early life factors in machine learning-based multimorbidity risk prediction

We thank the reviewers for their positive and insightful comments. The reviews have been of benefit to the manuscript, allowing us to greatly improve it. The following text lists each of the issues raised by the reviewers and the corresponding actions/answers (in blue). The track changes version of the manuscript highlights all modifications in blue and strikes out the removed sentences for easy identification.

Editor's comments

Thank you for submitting your manuscript to PLOS Digital Health. After careful consideration, we feel that it has merit but does not fully meet PLOS Digital Health's publication criteria as it currently stands. Therefore, we invite you to submit a revised version of the manuscript that addresses the points raised during the review process.

We sincerely thank the Editor for the opportunity to revise and resubmit our manuscript. Below, we provide detailed answers to the comments of the reviewers.

Reviewers' comments

Reviewer #1:

This study analyzes the contribution of ELFs to machine-learning-based risk prediction models for comorbid populations. You also utilized incorporation of life-course factors into risk models. Integrating a diverse range of physiological, psychological, and ELFs. I am wondering about integration of such a multilayered data to develop an AI model with a highly heterogeneous data??. The abstract is unstructured.

To address the concern of the reviewer, we have revised the abstract according to the journal's guidelines and conceptually structured it into background, methodology/principal findings, and conclusions. We also split the abstract into separate paragraphs to enhance readability. Additionally, we have included a sentence to clarify the ability of ML models to handle multilayered, heterogeneous data:

"To address the complexity of integrating diverse early-life and proximal factors, we leverage models capable of handling high-dimensional, heterogeneous data sources to enhance prediction accuracy in complex clinical populations."

Reviewer #2:

This paper uses a series of machine learning models and analyses to attempt to characterize the role of early life factors in multimorbidity risk prediction. Specifically, the

authors hope to use an examination of the role of these factors in predictive models to inform an understanding of how they might impact outcomes.

The paper is thorough and interesting, including a broad range of analyses that provide substantial understanding. Although there are a number of points that could use clarification, I did not identify any major methodological concerns.

We thank the reviewer for the positive feedback.

I am curious about one important point that was not addressed in the paper. If the goal was to address the role of early life factors, why start with machine learning models as the preferred analysis approach? Causal methods, such as propensity scoring or causal structural discovery, might yield some important insights, particularly regarding potential interactions between observed factors. The reasons for the selected approaches should be given and contrasted with alternative means of addressing questions of interest.

We thank the reviewer for this insightful comment regarding our analytical choices. Our study is designed to evaluate the contribution of early-life and proximal risk factors to the long-term prediction of CVD and diabetes within machine learning predictive models, particularly in comorbid populations. In addition to assessing their predictive utility, we also aim to understand the main drivers of model predictions, identifying which factors most influence risk estimation. Thus, our focus is not on analyzing causal effects of ELFs on disease development per se, but rather on investigating their role in enhancing predictive models and shaping the model's decision-making process.

To avoid any potential ambiguity, we have revised the manuscript text to clarify this distinction. Specifically, we have updated the sentence in the **Introduction** section from: **"In this paper, we leverage ML to investigate the impact of ELFs on CVD and diabetes, particularly in comorbid groups."** to: **"In this paper, we leverage ML to investigate the role of ELFs in predicting CVD and diabetes, particularly within comorbid groups."** (see page 3).

Additionally, we have strengthened the rationale for choosing machine learning over regression-based methods in the **Introduction** section (see page 3): **"It is important to distinguish that merely identifying statistical associations between variables, even if they are longitudinal and strong, does not necessarily translate into effective predictors for new observations, underscoring a gap in applying these findings to predictive modeling [16]."**

However, as the reviewer correctly noted, we had not explicitly contrasted our predictive approach with causal inference methods. To address this, we have added the following

sentence in the Introduction (see page 3, right after the previously mentioned sentence): **"In a similar vein, causal inference approaches (e.g., propensity scoring) can offer insights into mechanisms or intervention effects, but they are not designed to optimize predictive performance in complex, comorbid populations."**

Finally, we also mention as a limitation the inability of ML models to provide causal explanations (**Limitations** section, page 25): **"We also note that our models identify statistical associations between variables rather than causal pathways. Therefore, utilizing causal machine learning would be instrumental in exploring potential causal links between ELFs and multimorbidity outcomes in more diverse population-based samples."**

My other major concern with this paper is that the number of analyses is large and the volume of data is somewhat overwhelming. Some additional guidance, perhaps in terms of a workflow figure detailing the types of analyses and the goals presented might make the paper easier to read.

Following this helpful suggestion, we have added a new figure (Figure 1) that summarizes the analysis pipeline to improve clarity.

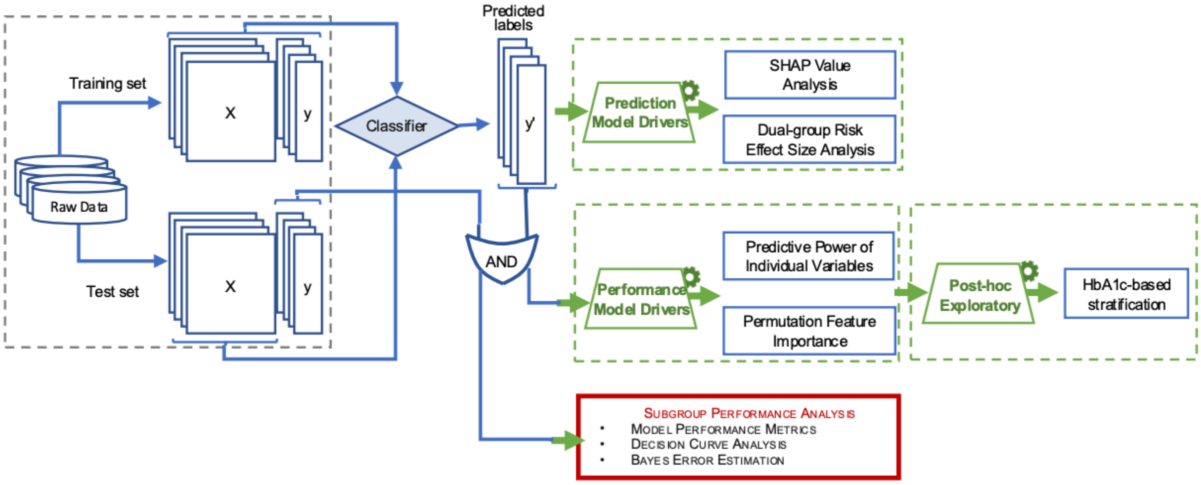

Fig 1. *Overview of the analytical framework characterizing the contribution of early-life and proximal factors in ML-based risk prediction of psycho-cardio-metabolic multimorbidity.*

Additionally, we include the following sentence (page 9) to guide readers:

"To summarize the analytical approach and goals addressed in this study, we provide a workflow diagram (Fig 1). This figure outlines the main analytical components:

subgroup performance analysis, characterization of performance and prediction model drivers, and post-hoc exploratory analyses."

A number of minor readability/comprehensibility issues arose during my reading of the paper:

1. In terms of selection of valuables (line 137-139), it might help to update Table S1 to indicate which factors are early life factors.

To address the concern of the reviewer, early-life factors are now clearly listed as a separate category in Table S1. We have also reordered the table to position ELFs as the top row, improving visibility and clarity. Additionally, we have also replaced the sentence in the main text where we point the reader to this table (page 5):

"Table S1 displays a comprehensive list of the 64 variables considered which are preprocessed into 83 features before modeling."

by:

"Table S1 lists the ELFs along with the other input variables among the 64 considered, grouped by category (e.g., sociodemographics, psychosocial factors, blood assays)."

---

2. Same lines, how are the 64 variables processed into 83 features? Details should be provided.

To clarify this point, we added the following sentence after the description of Table S1 in the manuscript (page 5):

"These variables were preprocessed into 83 features as a result of one-hot encoding applied to categorical variables with multiple levels."

---

3. Value of information - 207-217. Particularly since "value of information" has a specific meaning in many contexts, this term should be defined in more detail.

Thank you for the helpful comment. We have included the following sentence to clarify our use of the term at the beginning of the Materials and Methods subsection titled **"Value of information analysis"** (page 6):

"In this section, we examine the value of information provided by individual variables, including early-life factors. Specifically, we study their predictive utility for health outcomes and their role in shaping model decisions across subgroups."

---

4. Same section - the group-specific Bayes error estimation methods might not be familiar to all readers and should be described in a bit more detail.

To address this, we provide a more detailed explanation of group-specific Bayes error estimation in the Materials and Methods section. Specifically, we clarify that the minimal achievable error, aka Bayes error or irreducible noise, represents the portion of prediction error that no model can avoid (page 6):

"This minimal achievable error is also referred to as the Bayes error or irreducible noise, representing the portion of prediction error that cannot be eliminated by any model regardless of the learning algorithm. Estimating group-specific Bayes error provides insights into the inherent difficulty of disease prediction within each subgroup, helping to identify whether certain populations are fundamentally harder to forecast, independent of model choice."

We also explain how group-specific Bayes error provides insights into the inherent difficulty of prediction of disease outcomes within subgroups, and why we need to estimate its lower and upper bounds (page 6):

"We need to estimate the Bayes error since calculating the real value is unfeasible in general practice. We estimate its upper and lower bounds to narrow it down for each subgroup. The lower bound (Elow) represents the best-case minimal error achievable under ideal conditions, while the upper bound (Eup) reflects a worst-case estimate of the minimal error attainable even by the optimal classifier. The interval drawn with the upper and lower bounds for different subgroups can be compared. Non-overlapping intervals might indicate a different inherent difficulty of prediction for the groups."

---

5. I don't understand what is meant by the comment that "metrics like SHAP explain how variables contribute to performance but do not directly quantify their contribution to predictive performance. I assume that the point here is that SHAP is simply the result of multiple models that approximate the original model, but I am not quite sure. This somewhat cryptic statement should be replaced with additional clarification.

Thank you for highlighting this lack of clarity. SHAP values decompose the model's prediction f(x) into contributions from individual features. This allows us to interpret how each feature affects the output for a given instance. However, SHAP does not measure how much each feature impacts the model's performance (e.g., AUC, accuracy), that is, it does not evaluate how much each feature helps to predict better the true labels. To address this concern, we have replaced the referred sentence (pages 8-9):

"It is important to understand that metrics like SHAP explain how variables contribute to predictions but do not directly quantify their contribution to predictive performance."

by:

"It is important to understand that SHAP values help interpret the influence of each individual variable into the model's predictions (e.g., a variable taking a specific value might be associated in the model with a large predicted probability of disease). However, note that this cannot be interpreted as a measurement of how much each variable boosts the model's predictive performance (e.g., accuracy or AUC)."

---

6. The description of "Group-specific Bayes errors" is confusing, mentioning noise estimates that were not discussed when the Group-specific errors where introduced earlier. Although it's clear on a second read that "noise estimates" and "errors" are meant to be interchangeable, this paragraph would benefit from some clarification.

We acknowledge the confusion caused by the interchangeable use of "Bayes error" and "noise estimates." To clarify this relationship, we have added the following explanatory sentence to the **Materials and Methods** section, where Bayes error is first introduced (page 6):

"This minimal achievable error is also referred to as the Bayes error or irreducible noise, representing the portion of prediction error that cannot be eliminated by any model regardless of the learning algorithm."

---

7. Figure 1 - the caption needs to be edited. "Framming score" is not correct.

Typo corrected.

---

8. Figure 2 - the graphs as presented are a bit hard to read due to the difference in the range covered in the x- and y- axes. For example, the CVD graph seems to range from roughly 0.4- 0.6 on the x-axis and 0.45-0.63 on the y axis. This difference leads the equivalence (y=x) line to be skewed to the right, thus complicating the interpretation of which was better. A parallel presentation with the y=x line being a straight line from lower-left to upper right, would be much clearer.

We thank the reviewer for the suggestion and carefully considered the recommendation to adjust the axes to have identical ranges, so that the y = x line would appear as a perfect 45-degree diagonal from the bottom-left to the top-right. However, we found that strictly enforcing identical x- and y-axis ranges led to a significant compression of the points into a narrow area, which reduced the granularity and made it harder to distinguish important differences between variables.

To address the reviewer's concern while preserving detail, we adjusted the x-axis limits in the CVD plot to better center the data and improve interpretability. Specifically, we removed three variables with AUC-ROC < 0.47 in the comorbid group, which helped reduce visual compression. These changes improve the clarity of the y=x reference line and enhance the comparability of variable performance across groups. This adjustment is also noted in the updated figure caption (page 15), which states: "**To improve visual clarity, three variables with AUC-ROC < 0.47 in the comorbid group were omitted from the CVD plot (a).**"

---

9. The discussion of HbA1c's role in diabetes prediction reads more like methods than results. Perhaps this should be moved to the methods section.

We thank the reviewer for the suggestion. We have moved the description of the HbA1c stratified analysis, including subgroup definitions and model evaluations, from the Results section to the Materials and Methods section (see **"Post-hoc analysis based on dominant performance drivers"**, page 9). The Results section now focuses solely on the analysis outcomes, with a brief introductory sentence linking to the Methods (pages 19-20): **"As outlined in Section Materials and Methods, a post-hoc stratified analysis was performed to evaluate the role of HbA1c in diabetes prediction within the comorbid population, following its identification as a dominant performance driver (Table 3)."**

---

10. Finally, there are a great many results in this paper that are hard to interpret. Some sort of tabular summary and/or clearer delineation of the topics in the discussion would make the paper more readable and the conclusions more digestible.

Following the suggestion of the reviewer, we introduced multiple subdivisions in the **Results** section to clearly delineate different analyses. We also separated lengthy paragraphs into smaller, more focused ones, each emphasizing a specific finding or interpretation, to make the narrative more digestible.
