# Supplementary material for: Characterizing the role of early life factors in machine learning-based multimorbidity risk prediction
Source: PLOS Digit Health. 2025 Aug 18;4(8):e0000982. doi: 10.1371/journal.pdig.0000982 (PMC12360575; doi:10.1371/journal.pdig.0000982)
Supplement: Attachment — Submitted filename: Response_to_Reviewers_auresp_2.pdf [file pdig.0000982.s013.pdf]

RESPONSE LETTER TO THE REVIEWERS' COMMENTS
Ms. Ref. No.: PDIG-D-25-00125

Characterizing the role of early life factors in machine learning-based multimorbidity risk prediction

We thank the reviewers for their positive and insightful comments. The reviews have been of benefit to the manuscript, allowing us to greatly improve it. The following text lists each of the issues raised by the reviewers and the corresponding actions/answers (in blue). The track changes version of the manuscript highlights all modifications in blue and strikes out the removed sentences for easy identification.

Editor's comments

Thank you for submitting your manuscript to PLOS Digital Health. After careful consideration, we feel that it has merit but does not fully meet PLOS Digital Health's publication criteria as it currently stands. Therefore, we invite you to submit a revised version of the manuscript that addresses the points raised during the review process.
We sincerely thank the Editor for the opportunity to revise and resubmit our manuscript. Below, we provide detailed answers to the comments of the reviewers.

Reviewers' comments

Reviewer #2:

This revised paper addresses most of my concerns. Thanks to the authors for their attention to comments. I have two outstanding concerns:

1 I appreciate your change in language regarding "Value of information", but I find the revision to be inadequate. "Value of information" is an accepted phrase in the literature, involving structured methods for determining the marginal value of information in decision-making. See, for example, Heath, et al. 2017 (DOI 10.1177/0272989X176976) for an overview. As this paper does not use any of those methods, calling this section a VOI analysis is inappropriate. I suggest rephrasing this section, perhaps labeling it as "analysis of contributions of predictive factors".

Thank you for pointing out the conflicting use of the term "Value of Information." To address this, we have renamed the section to "**Variable Contribution Analysis**" to avoid misunderstanding and reflect more accurately our methodology. Accordingly, we have also replaced the introductory sentence (see page 6): *"In this section, we examine the value of information provided by individual variables, including early-life factors."* with: **"In this section, we examine the contribution of individual variables to model performance and decision-making, including early-life factors. Specifically, we study their predictive utility for health outcomes and their role in shaping model decisions across subgroups."**

2 I appreciate the response regarding the graphs comparing the ROCs for comorbid and non-comorbid groups, but I believe the groups are still misleading. I suggest revising both graphs to have 0.5 as the lowest point for both the x- and y-axes. This would provide the desired 45 degree angle while avoiding both the compression that might be associated with showing the whole range and the difficulties of comparison created by having the range of values on the axes differ for the two graphs.

We thank the reviewer for this constructive suggestion. In the revised version of Fig. 3, we have standardized the axis range across both the CVD and diabetes plots to ensure consistent visual scaling and to facilitate better interpretability of the diagonal 45-degree reference line. Specifically, the axis limits were chosen based on the pooled AUC-ROC values from both tasks: the lower bound was set to the minimum value observed among Early-life factor (ELF) variables—given their central importance in our study—and the upper bound was set at the 99th percentile of the combined AUC-ROC distribution. This approach maintains all ELF variables within view, avoids excessive compression of the plots, and supports more interpretable visual comparisons. The full set of variables, including those beyond the zoomed-in range, is shown in the supplementary material (Fig. S4) for completeness.

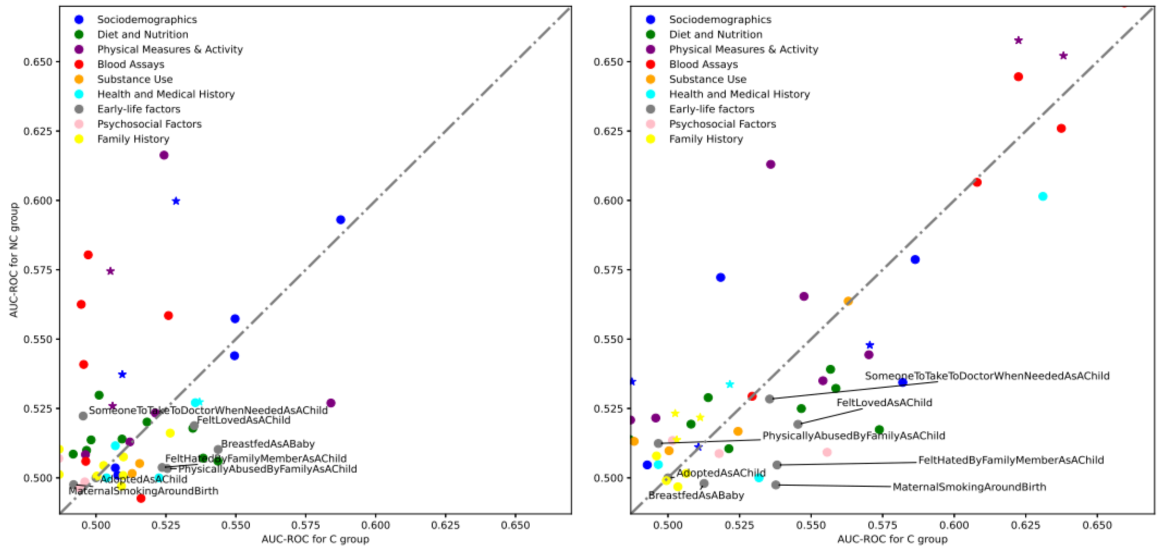

(a) XGB CVD

(b) XGB Diabetes

Fig 3. Predictive ability of individual variables for (a) CVD and (b) diabetes prediction. Each plot compares AUC-ROC performance in the comorbid group (x-axis) vs performance in the non-comorbid (y-axis) group. Each point represents an XGB model learned with a single feature. Colors differentiate models learned with a variable of different type. The star marker indicates models learned with a variable used by the benchmarks. The dotted grey line indicates equal performance in both groups. Deviation from this line allows for identifying variables that have a stronger predictive impact on one group over the other.

We have added two sentences to the manuscript text according to this change (see page 12): "**To improve visual interpretability and enable consistent comparison, the**

horizontal and vertical ranges are delimited to a lower bound based on the minimum value observed among ELF variables and an upper bound at the 99th percentile of the distribution of values from both conditions." and "**The complete distribution of AUC-ROC values for all the features is available in Fig S4.**"

Two minor points

1 Please provide version numbers for libraries mentioned in the methods section.

See below.

2 Consider making the code available as open source.

We thank the reviewer for these helpful suggestions. To address both points, we have made all implementation details, including code and library versions, publicly available at *https://github.com/ngoc-vien-dang/ELF*. Accordingly, we added the following sentence to the Materials and Methods section (see page 9): **"All implementation details, including code and library versions, are available at https://github.com/ngoc-vien-dang/ELF."**

Reviewer #3:

The objective of this study is to study how machine learning can investigate the role of early-life factors in predicting cardiovascular disease and diabetes. This essay is an attempt to contribute to the predictive model literature by showing how to represent and integrate early-life factors in model development and of the importance of doing so. This is challenging due to the complexity of the relevant variables, their nonlinear interactions, and the assumptions a model might make. The hope is to provide insights that enhance the accuracy of existing risk prediction algorithms, specifically tailored for individuals with comorbid conditions.

I found the essay intriguing, very well written, and admirably detailed in its methodological description and findings. Quite frankly, this is a "big" article. I'm not trained in the data science dimensions that inform the study design so I must defer to other reviewers who can comment on their validity. I can say, though, that the effort to study early-life factors seems to me quite valuable, such that even if readers find material to criticize, the issues presented in this paper should be of value to the digital health community. Nevertheless, it would seem to me that the only persons with enough background to critique the methodology of the study

would be data science specialists. Consequently, their review of this study is of quintessential importance.

Three drawbacks of the study are already mentioned by the authors. The first, that the model was normed using a United Kingdom database, with over 96% of participants being white. Another problem might be the accuracy of the data being entered, especially as it reports on early life factors. One is especially worried about data that are not only inaccurate but missing. A third factor is simply the plethora of variables programmed into the model. I suppose one can question whether that degree of detail is necessary or really contributes to the model's predictive accuracy—although that question would obviously be addressed in future test applications of the model.

Ultimately, I found this essay very impressive in virtually every way. Should other reviewers find the methodology and findings acceptable, I would strongly endorse the article's acceptance and eventual publication.

Thank you for the positive assessment of our study. We also acknowledge the reviewer's thoughtful reflection on the study's limitations, which are discussed in the manuscript. We agree that continued validation, particularly through future applications in more diverse and representative datasets, will be important for advancing this line of research.
